# Supplementary material for: Reliability of resting-state EEG modulation by continuous and intermittent theta burst stimulation of the primary motor cortex: a sham-controlled study
Source: Sci Rep. 2023 Nov 2;13:18898. doi: 10.1038/s41598-023-45512-6 (PMC10622440; doi:10.1038/s41598-023-45512-6)
Supplement: Supplementary file 1 — Supplementary Information. [file 41598_2023_45512_MOESM1_ESM.pdf]

Supplementary material

Reliability of resting-state EEG modulation by continuous and intermittent theta burst stimulation of the primary motor cortex: A sham-controlled study

Andrei Rodionov [1, 2], Recep A. Ozdemir [1, 2], Christopher S.Y. Benwell [3], Peter J. Fried [1, 2], Pierre Boucher [1], Davide Momi [1, 2, 4], Jessica M. Ross [1, 2, 5, 6], Emiliano Santarnecchi [2, 7, ^], Alvaro Pascual-Leone [2, 8, ^], Mouhsin M. Shafi [1, 2, *, ^]

[1] Berenson-Allen Center for Noninvasive Brain Stimulation, Department of Neurology, Beth Israel Deaconess Medical Center, Boston, MA, USA
[2] Department of Neurology, Harvard Medical School, Boston, MA, USA
[3] Division of Psychology, School of Humanities, Social Sciences and Law, University of Dundee, Dundee, UK
[4] Krembil Centre for Neuroinformatics, Centre for Addiction & Mental Health, Toronto
[5] Veterans Affairs Palo Alto Healthcare System, and the Sierra Pacific Mental Illness, Research, Education, and Clinical Center, Palo Alto, CA, USA
[6] Department of Psychiatry and Behavioral Sciences, Stanford Medical School, Stanford, CA, USA
[7] Precision Neuroscience & Neuromodulation Program, Gordon Center for Medical Imaging, Massachusetts General Hospital, Harvard Medical School, Boston, MA, USA;
[8] Hinda and Arthur Marcus Institute for Aging Research, Deanna and Sidney Wolk Center for Memory Health, Hebrew Senior Life, Boston, MA, USA

* Corresponding authors: mshafi@bidmc.harvard.edu
^ Shared senior authorship

Supplementary methods

MRI acquisition

A T1-weighted structural MR image was obtained for each subject with a 3T MRI scanner (GE Healthcare, Ltd., United Kingdom) and used for TMS navigation. MRIs were taken using a 3D spoiled gradient echo sequence (166 axial slices, 240-mm isotropic field-of view, voxel size 0.937 × 0.937 × 1 mm, flip angle = 15°, TE/TR ≥ 2.9/6.9 ms, duration ≥ 432 s).

Determination of motor hotspot, resting and active motor threshold

Participants were comfortably seated in an armchair and were instructed to keep their eyes open and their muscles relaxed during the experiments. Monophasic single-pulse TMS (spTMS) was delivered to the left primary motor cortex (M1) with a MagPro Cool-B65 A/P figure-of-eight coil (75mm outer wing diameter) and a MagPro X100 stimulator (MagVenture A/S, Farum, Denmark). Brainsight™ TMS Frameless Navigation system (Rogue Research Inc., Montreal, Canada) was used for real time monitoring of accurate and reproducible coil positioning within and across sessions. Motor hotspot, resting and active motor thresholds (RMT and AMT) were found in each participant for the first dorsal interosseous (FDI) muscle. For motor hotspot search the coil was placed over the hand area of M1 and oriented to induce a posterior–anterior current perpendicular to the central sulcus. The hotspot was defined as the location within M1 where TMS induced the largest and consistent MEPs. RMT was defined as the lowest stimulator intensity expressed as percentage of maximum stimulator output (%MSO) to elicit MEPs of at least 50 μV peak-to-peak amplitude in 5 out of 10 consecutive trials in relaxed target muscle. AMT was defined as the lowest %MSO to elicit MEPs of at least 200 μV in 5 out of 10 consecutive trials in the voluntary contracted target muscle. Participants wore earplugs throughout the stimulation sessions for hearing protection.

Motor evoked potentials (MEPs)

Motor evoked potentials (MEPs) were recorded from the first dorsal interosseous (FDI) muscle of the right hand. In pre-TBS 120 single-pulse TMS (spTMS) were given and corresponding MEPs were recorded. After that, one of three TBS protocols was applied, followed by blocks of 60 spTMS. For the purpose of this study we selected and analyzed only MEPs recorded within the blocks at 5, 20 and 30 min after the TBS. Mean MEP amplitudes for each post-TBS time-point were expressed as the percentage change from pre-TBS. For details of MEP analysis see the previous study [1].

Supplementary results

TBS-induced modulation of EEG relative power in the right hemisphere and absolute power in both hemispheres

The results of LMMs of relative power on ROI Right revealed no significant main effects of factor Time in V1 and V2 (all F-values < 3.00, all p-values > 0.05 (corr), partial eta < 0.15, Supplementary Table S1). The results of LMMs of absolute power on ROI Left and Right revealed a single main effect of factor Time in the ROI Left in cTBS V2 in beta 1 band which didn't survive correction for multiple comparison (F-value = 2.76, p-values > 0.05 (corr), partial eta < 0.12, Supplementary Table S2).

Whole-brain analysis of TBS neuromodulatory effects on EEG absolute power

The analysis of absolute power didn't identify any significant clusters.

Modulation of corticospinal excitability

We briefly describe the results of MEP analysis previously published by [1]. MEP amplitudes were increased by iTBS and sham TBS in V1 and V2 (Figure S2). cTBS had facilitatory effect on MEPs in V2. For details of results of MEP analysis see the previous study [1].

Comparisons of EEG modulation between visits and test-retest reliability of TBS effects

The results of LMM of relative power in the ROI Right yielded no significant main effect of factor Visit (all F-values < 3.00, all p-values > 0.05 (corr.), partial eta < 0.15, Table S4). The results of LMM of absolute power in the ROI Left and ROI Right yielded no significant main effect of factor Visit (all F-values < 2.00, all p-values > 0.05 (corr.), partial eta < 0.10, Table S5). Overall, reliability of modulation of relative power in ROI Right and absolute power in both ROIs was low-to-moderate (Cronbach's a < 0.75, Table S6). High between-visit reliability of modulation of relative power was obtained in the ROI Right in cTBS at T15 in beta 1 band (Cronbach's a=0.83, Table S6) and in sham TBS also in beta 1 band at T15 (Cronbach's a=0.78, Table S6) and T25 (Cronbach's a=0.98, Table S6). Modulation of absolute EEG power was highly consistent between two visits in the ROI Right after sham TBS in alpha band at T25 (Cronbach's a=0.85, Table S6).

Contingency analysis of individual TBS-induced changes

The contingency analysis revealed that distributions of change types were not statistically different between the visits (χ^2(3, N=43-45)<7.00, p>0.05 (corr.), see Supplementary table S7). The amount of participants who exhibited significant post-TBS change varied between protocols, visits and frequency bands being on average 76±13% (range 52 and 95%). There was no any clear tendency towards increase or decrease of power attributable to TBS protocol or frequency band. On average 65% of participants exhibited different change types in V1 and V2 (Figure S3). Like the results of analysis of relative power, both active and sham TBS protocols led to large inter-subject variability of types of EEG changes across the visits (cTBS – 64±10%, iTBS – 79±0%, sham TBS – 53±18%, average across alpha and beta1 bands).

Comparison of EEG modulation by active and sham TBS protocols

The results of LMMs of relative power in ROI Right yielded significant main effect of factor Protocol in beta 1 band in V1 which didn't survive correction for multiple comparison (F-value = 4.69, p-values > 0.05 (corr), partial eta = 0.05). The results of LMMs of absolute power in ROI Left and ROI Right also yielded significant main effects of factor Protocol in beta 1 band in V2 which didn't survive correction for multiple comparison (F-value = 4.81, p-values > 0.05 (corr), partial eta = 0.05, and F-value = 3.31, p-values > 0.05 (corr), partial eta = 0.04 correspondingly).

Relationship between EEG spectral power and corticospinal excitability

We performed analysis of relationships between modulation of EEG power in alpha and beta 1 bands in the non-targeted right hemisphere (ROI Right) and corticospinal excitability of the contralateral side by correlating EEG changes and MEPs in those protocols and time points where correlations in the targeted left hemisphere (ROI Left) were found. There was a negative correlation between modulation of relative power in the ROI Right in beta 1 band at T15 and MEP modulation in the left hemisphere at T20 in iTBS V1 (Pearson's r = -0.52, p < 0.05). Modulation of relative power in beta 1 band at T0 in the ROI Right

positively correlated with MEP changes in the left hemisphere at T5 in sham V1 (Pearson's $r = 0.60$, $p < 0.05$) and in sham V2 (Pearson's $r = 0.53$, $p = 0.01$).We also found positive correlation between modulation of absolute EEG power in the ROI Right in alpha band at T15 and MEP modulation at T20 in iTBS V1 (Pearson's $r = 0.48$, $p < 0.05$).

References

[1] P. O. Boucher *et al.*, “Sham-derived effects and the minimal reliability of theta burst stimulation,” *Sci Rep*, vol. 11, no. 1, Dec. 2021, doi: 10.1038/s41598-021-98751-w.

Supplementary figures and tables

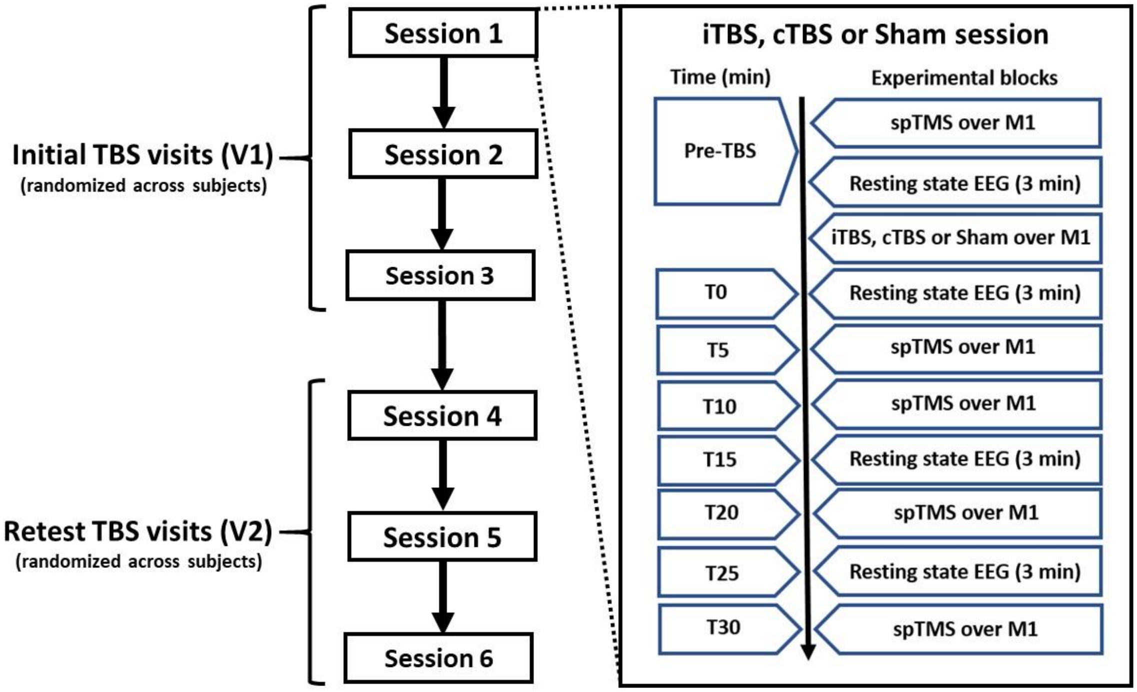

Supplementary Figure S1. Experimental design. spTMS – single pulse TMS, M1 – the primary motor cortex

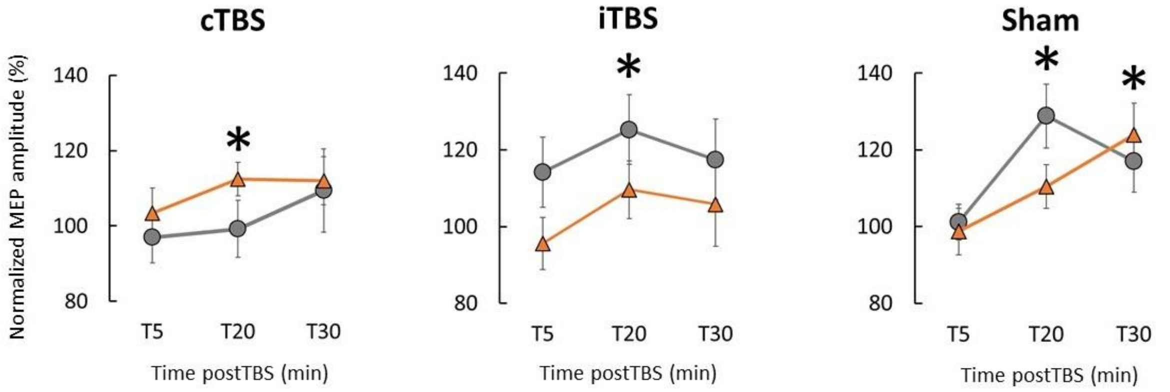

Supplementary Figure S2. Modulation of corticospinal excitability by cTBS, iTBS and sham TBS. Gray round markers - group means of normalized MEP amplitudes (post-TBS/pre-TBS ratios) at 5, 20 and 30 min post-TBS in the initial visit (V1), orange triangles - corresponding values in the retest visit (V2). Error bars – standard error of the mean. Asterisks denote significant MEP changes in comparison to 100% pre-TBS ($p < 0.05$). Significant MEP facilitation by iTBS in V2 occurred at T10 (not shown).

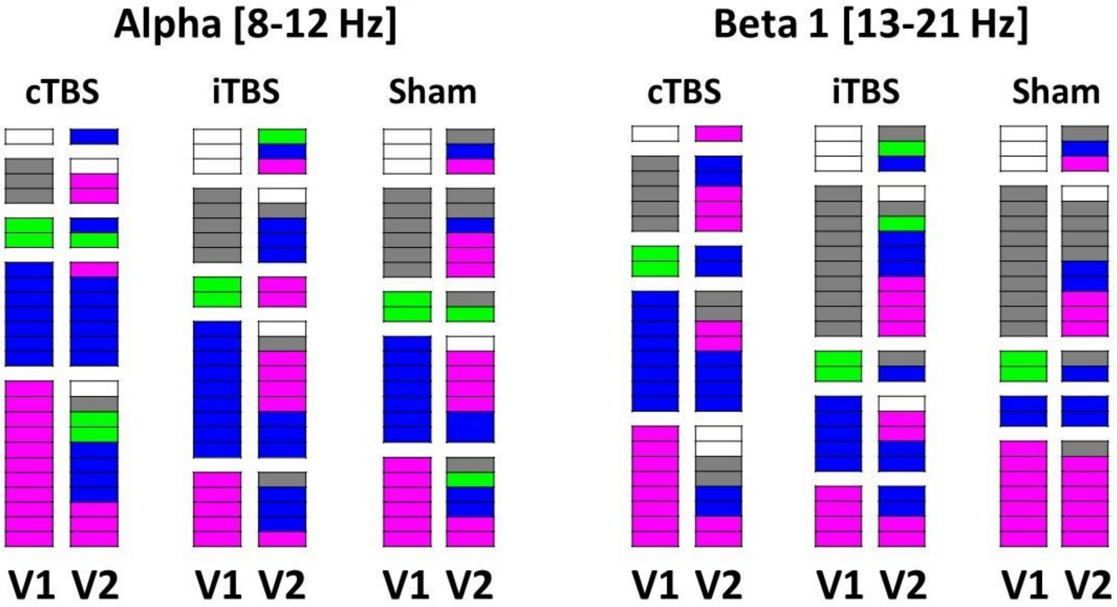

Supplementary figure S3. Conversion of EEG change types in each participant in alpha and beta 1 bands in the initial (V1) and retest (V2) visits. Different types of change are given in different colors: magenta - increase of absolute power in post-TBS in comparison to pre-TBS, blue – decrease of power, green - mixed change including increase and decrease at different post-TBS time points, grey - no change, white – missing data.

Supplementary Table S1. Results of linear mixed model analysis of TBS neuromodulatory effects on relative EEG power in ROI Left and ROI Right in the initial (V1) and retest (V2) visits

Protocol	Visit	Frequency	ROI Left						ROI Right					
			N parm	DF	DF Den	F-value	p-value (uncorr)	Partial eta	N parm	DF	DF Den	F-value	p-value (uncorr)	Partial eta
cTBS	V1	Alpha	3	3	65.02	2.322	0.083	0.10	3	3	65.04	1.737	0.168	0.07
		Beta 1	3	3	65.02	1.953	0.130	0.08	3	3	65.03	2.635	0.057	0.11
	V2	Alpha	3	3	63.00	0.415	0.743	0.02	3	3	63.00	0.327	0.806	0.02
		Beta 1	3	3	63.00	1.249	0.299	0.06	3	3	63.00	0.817	0.489	0.04
iTBS	V1	Alpha	3	3	58.04	0.497	0.686	0.03	3	3	58.04	0.200	0.896	0.01
		Beta 1	3	3	58.05	0.102	0.959	0.01	3	3	58.05	0.157	0.925	0.01
	V2	Alpha	3	3	65.03	0.786	0.506	0.04	3	3	65.03	0.425	0.736	0.02
		Beta 1	3	3	65.02	0.887	0.452	0.04	3	3	65.03	1.022	0.389	0.05
Sham	V1	Alpha	3	3	57.16	0.839	0.478	0.04	3	3	57.13	0.782	0.509	0.04
		Beta 1	3	3	57.03	0.048	0.986	0.00	3	3	57.03	0.242	0.867	0.01
	V2	Alpha	3	3	64.00	0.092	0.964	0.00	3	3	64.00	0.080	0.971	0.00
		Beta 1	3	3	64.10	0.259	0.854	0.01	3	3	64.10	0.049	0.986	0.00

DF – degrees of freedom for the numerator, DF Den – degrees of freedom for the denominator, p-value (uncorr) – uncorrected p-value, ROI Left and ROI Right – regions of interest in the left and right hemisphere

Supplementary Table S2. Results of linear mixed model analysis of TBS neuromodulatory effects on absolute EEG power in the initial (V1) and retest (V2) visits

Protocol	Visit	Frequency	ROI Left						ROI Right					
			N parm	DF	DF Den	F-value	p-value (uncorr)	Partial eta	N parm	DF	DF Den	F-value	p-value (uncorr)	Partia l eta
cTBS	V1	Alpha	3	3	65.00	1.561	0.207	0.07	3	3	65.03	1.490	0.225	0.06
		Beta 1	3	3	65.00	0.692	0.560	0.03	3	3	65.01	0.751	0.526	0.03
	V2	Alpha	3	3	63.00	1.977	0.127	0.09	3	3	63.00	1.400	0.251	0.06
		Beta 1	3	3	63.00	2.757	**0.049**	0.12	3	3	63.00	2.244	0.092	0.10
iTBS	V1	Alpha	3	3	58.02	0.056	0.983	0.00	3	3	58.01	0.132	0.941	0.01
		Beta 1	3	3	58.00	0.520	0.670	0.03	3	3	58.00	0.863	0.466	0.04
	V2	Alpha	3	3	65.03	0.217	0.885	0.01	3	3	65.02	0.123	0.946	0.01
		Beta 1	3	3	65.02	0.209	0.890	0.01	3	3	65.01	0.127	0.944	0.01
Sham	V1	Alpha	3	3	57.06	1.046	0.379	0.05	3	3	57.06	0.929	0.433	0.05
		Beta 1	3	3	57.01	1.176	0.327	0.06	3	3	57.00	1.330	0.274	0.07
	V2	Alpha	3	3	64.08	0.087	0.967	0.00	3	3	64.08	0.087	0.967	0.00
		Beta 1	3	3	64.04	0.923	0.435	0.04	3	3	64.03	0.506	0.679	0.02

DF – degrees of freedom for the numerator, DF Den – degrees of freedom for the denominator, p-value (uncorr) – uncorrected p-value, ROI Left and ROI Right – region of interest in the left and right hemisphere, bold font – significant p-value before correction.

Supplementary Table S3 Results of linear mixed model and reliability analysis of relative and absolute EEG power at the baseline (pre-TBS) in the ROI Left and Right across six stimulation sessions

Frequency	ROI Left							ROI Right						
	N parm	DF	DF Den	F-value	p-value (uncorr)	Partial eta	Cronbach's alpha	N parm	DF	DF Den	F-value	p-value (uncorr)	Partial eta	Cronbach's alpha
	Relative power							Relative power						
Alpha	5	5	104.2	1.033	0.402	0.05	0.98	5	5	104.2	0.348	0.883	0.02	0.97
Beta 1	5	5	104.1	0.292	0.917	0.01	0.99	5	5	104.1	0.202	0.961	0.01	0.99
	Absolute power							Absolute power						
Alpha	5	5	104.1	1.353	0.248	0.06	0.98	5	5	104.1	0.374	0.866	0.02	0.98
Beta 1	5	5	104.1	1.454	0.211	0.07	0.98	5	5	104.0	0.318	0.901	0.02	0.98

DF – degrees of freedom for the numerator, DF Den – degrees of freedom for the denominator, p-value (uncorr) – uncorrected p-value, ROI Left and ROI Right – region of interest in the left and right hemisphere

Supplementary table S4. Results of linear mixed model analysis of TBS-induced modulation of relative EEG power in ROI Left and ROI Right across visits

Protocol	Frequency	Time postTBS	ROI Left						ROI Right					
			N parm	DF	DF Den	F-value	p-value (uncorr)	Partial eta	N parm	DF	DF Den	F-value	p-value (uncorr)	Partial eta
cTBS	Alpha	T0	1	1	23.24	0.812	0.377	0.03	1	1	22.84	0.127	0.725	0.01
		T15	1	1	21.81	0.401	0.533	0.02	1	1	22.30	0.469	0.501	0.02
		T25	1	1	22.33	0.181	0.675	0.01	1	1	22.49	0.079	0.782	0.00
	Beta 1	T0	1	1	22.58	1.003	0.327	0.04	1	1	23.36	0.013	0.910	0.00
		T15	1	1	22.43	0.094	0.762	0.00	1	1	21.64	1.908	0.181	0.08
		T25	1	1	22.96	1.213	0.282	0.05	1	1	23.23	2.671	0.116	0.10
iTBS	Alpha	T0	1	1	20.41	5.251	**0.033**	0.20	1	1	21.87	0.314	0.581	0.01
		T15	1	1	24.49	0.016	0.902	0.00	1	1	24.35	0.140	0.711	0.01
		T25	1	1	23.30	1.341	0.259	0.05	1	1	23.61	0.376	0.546	0.02
	Beta 1	T0	1	1	22.84	0.287	0.597	0.01	1	1	21.88	0.002	0.966	0.00
		T15	1	1	24.66	0.020	0.888	0.00	1	1	24.85	0.174	0.680	0.01
		T25	1	1	24.28	0.586	0.451	0.02	1	1	24.34	0.586	0.451	0.02
Sham	Alpha	T0	1	1	19.57	0.155	0.698	0.01	1	1	21.55	0.316	0.580	0.01
		T15	1	1	21.53	0.032	0.859	0.00	1	1	23.08	0.003	0.961	0.00
		T25	1	1	23.41	0.527	0.475	0.02	1	1	24.14	0.779	0.386	0.03
	Beta 1	T0	1	1	24.13	0.414	0.526	0.02	1	1	24.49	0.000	0.989	0.00
		T15	1	1	24.34	0.006	0.938	0.00	1	1	24.38	0.218	0.645	0.01
		T25	1	1	24.31	0.019	0.892	0.00	1	1	23.91	0.015	0.903	0.00

DF – degrees of freedom for the numerator, DF Den – degrees of freedom for the denominator, p-value (uncorr) – uncorrected p-value, ROI Left and ROI Right – region of interest in the left and right hemisphere

Supplementary table S5. Results of linear mixed model analysis of TBS-induced modulation of absolute EEG power in ROI Left and ROI Right across visits

Protocol	Frequency	Time postTBS	ROI Left						ROI Right					
			N parm	DF	DF Den	F-value	p-value (uncorr)	Partial eta	N parm	DF	DF Den	F-value	p-value (uncorr)	Partial eta
cTBS	Alpha	T0	1	1	22.93	0.549	0.466	0.02	1	1	23.32	0.361	0.554	0.02
		T15	1	1	21.90	0.114	0.738	0.01	1	1	22.29	0.015	0.905	0.00
		T25	1	1	23.39	0.003	0.960	0.00	1	1	23.36	0.004	0.950	0.00
	Beta 1	T0	1	1	22.16	0.000	0.987	0.00	1	1	22.88	0.538	0.471	0.02
		T15	1	1	22.94	1.299	0.266	0.05	1	1	22.61	1.435	0.243	0.06
		T25	1	1	23.18	0.700	0.412	0.03	1	1	22.97	1.222	0.281	0.05
iTBS	Alpha	T0	1	1	22.78	0.386	0.541	0.02	1	1	22.73	0.094	0.763	0.00
		T15	1	1	23.37	0.000	0.989	0.00	1	1	23.33	0.120	0.732	0.01
		T25	1	1	24.15	0.021	0.887	0.00	1	1	24.03	0.217	0.646	0.01
	Beta 1	T0	1	1	21.96	0.313	0.581	0.01	1	1	22.60	0.789	0.384	0.03
		T15	1	1	20.27	0.061	0.808	0.00	1	1	17.71	0.427	0.522	0.02
		T25	1	1	22.96	0.351	0.560	0.02	1	1	23.76	0.595	0.448	0.02
Sham	Alpha	T0	1	1	22.67	0.796	0.382	0.03	1	1	23.24	0.058	0.811	0.00
		T15	1	1	23.25	0.237	0.631	0.01	1	1	23.70	0.020	0.890	0.00
		T25	1	1	24.32	0.083	0.775	0.00	1	1	24.05	0.117	0.735	0.00
	Beta 1	T0	1	1	20.71	0.694	0.415	0.03	1	1	19.28	0.426	0.522	0.02
		T15	1	1	21.56	1.578	0.223	0.07	1	1	21.35	0.486	0.493	0.02
		T25	1	1	22.91	0.062	0.805	0.00	1	1	22.66	0.006	0.942	0.00

DF – degrees of freedom for the numerator, DF Den – degrees of freedom for the denominator, p-value (uncorr) – uncorrected p-value, ROI Left and ROI Right – region of interest in the left and right hemisphere

Supplementary Table S6. Cronbach's alpha coefficients of consistency of TBS-induced EEG modulation across visits

Protocol	Frequency band	Time postTBS	Relative power	Absolute power	
			ROI Right	ROI Left	ROI Right
cTBS	Alpha	T0	0.504	0.045	0.028
		T15	0.677	0.744	0.691
		T25	0.668	0.216	0.304
	Beta 1	T0	0.174	0.021	0.303
		T15	0.829	0.262	0.588
		T25	0.378	0.296	0.498
iTBS	Alpha	T0	0.067	0.286	0.276
		T15	0.748	0.475	0.499
		T25	0.310	0.443	0.436
	Beta 1	T0	0.332	0.127	0.068
		T15	0.531	0.365	0.509
		T25	0.494	0.338	0.392
Sham	Alpha	T0	0.027	0.262	0.346
		T15	0.006	0.067	0.493
		T25	0.712	0.691	0.852
	Beta 1	T0	0.684	0.460	0.601
		T15	0.777	0.674	0.390
		T25	0.980	0.346	0.258

Supplementary Table S7. Results of contingency analysis of individual TBS-induced changes

Protocol	Frequency	Relative power					Absolute power				
		N	DF	Pearson chi-square	p-value (uncorr.)	Type conversion (%)	N	DF	Pearson chi-square	p-value (uncorr.)	Type conversion (%)
cTBS	Alpha	45	3	3.989	0.263	76	45	3	3.966	0.265	57
	Beta 1	45	3	1.752	0.417	67	45	3	2.312	0.510	71
iTBS	Alpha	43	3	3.354	0.340	79	43	3	1.556	0.669	79
	Beta 1	43	3	2.244	0.523	63	43	3	6.226	0.101	79
Sham	Alpha	44	3	0.470	0.925	65	44	3	1.079	0.782	65
	Beta 1	44	3	0.369	0.832	55	44	3	4.978	0.173	40

N – number of samples, DF – degrees of freedom, p-value (uncorr.) – uncorrected p-value, type conversion – number of participants demonstrating different types of EEG change across two visits.

Supplementary table S8. Numerical proportions of types of TBS-induced changes of absolute EEG power in ROI Left in the initial (V1) and retest (V2) visits

Protocol	Visit	Alpha				Beta 1			
		Increase of power	Decrease of power	Mixed change	No change	Increase of power	Decrease of power	Mixed change	No change
cTBS	V1	48	30	9	13	35	35	9	22
	V2	27	55	14	5	36	45	0	18
iTBS	V1	24	43	10	24	19	24	10	48
	V2	36	45	5	14	36	41	9	14
Sham	V1	29	33	10	29	33	10	10	48
	V2	43	26	9	22	43	26	0	30

Supplementary table S9. Results of linear mixed model analysis of TBS-induced modulation of relative EEG power in ROI Left and ROI Right across active and sham TBS protocols

Visit	Frequency	Factor	ROI Left						ROI Right					
			N parm	DF	DF Den	F-value	p-value (uncorr)	Partial eta	N parm	DF	DF Den	F-value	p-value (uncorr)	Partial eta
V1	Alpha	Protocol	2	2	166.6	2.018	0.136	0.02	2	2	165.1	1.595	0.206	0.02
		Protocol x Time	4	4	157.0	0.815	0.518	0.02	4	4	155.6	0.417	0.796	0.01
	Beta 1	Protocol	2	2	167.3	2.104	0.125	0.02	2	2	170.2	4.685	**0.011**	0.05
		Protocol x Time	4	4	158.8	0.353	0.841	0.01	4	4	159.9	0.302	0.876	0.01
V2	Alpha	Protocol	2	2	173.9	0.783	0.459	0.01	2	2	175.1	0.077	0.926	0.00
		Protocol x Time	4	4	169.3	0.149	0.963	0.00	4	4	170.1	0.140	0.967	0.00
	Beta 1	Protocol	2	2	174.9	1.462	0.235	0.02	2	2	176.1	1.414	0.246	0.02
		Protocol x Time	4	4	170.1	0.404	0.806	0.01	4	4	170.6	0.149	0.963	0.00

DF – degrees of freedom for the numerator, DF Den – degrees of freedom for the denominator, p-value (uncorr) – uncorrected p-value, ROI Left and ROI Right – region of interest in the left and right hemisphere

Supplementary table S10. Results of linear mixed model analysis of TBS-induced modulation of absolute EEG power in ROI Left and ROI Right across active and sham TBS protocols

Visit	Frequency	Factor	ROI Left						ROI Right					
			N parm	DF	DF Den	F-value	p-value (uncorr)	Partial eta	N parm	DF	DF Den	F-value	p-value (uncorr)	Partial eta
V1	Alpha	Protocol	2	2	168.5	1.060	0.349	0.01	2	2	167.6	1.834	0.163	0.02
		Protocol x Time	4	4	158.5	0.714	0.584	0.02	4	4	158	0.623	0.647	0.02
	Beta 1	Protocol	2	2	165.2	0.808	0.448	0.01	2	2	164.3	1.293	0.277	0.02
		Protocol x Time	4	4	158.4	0.612	0.655	0.02	4	4	158.3	0.967	0.428	0.02
V2	Alpha	Protocol	2	2	174.6	2.948	0.055	0.03	2	2	175.8	1.617	0.201	0.02
		Protocol x Time	4	4	170.4	0.311	0.870	0.01	4	4	170.7	0.191	0.943	0.00
	Beta 1	Protocol	2	2	173.3	4.813	**0.009**	0.05	2	2	173.6	3.308	**0.039**	0.04
		Protocol x Time	4	4	170.1	0.768	0.547	0.02	4	4	169.9	0.358	0.839	0.01

DF – degrees of freedom for the numerator, DF Den – degrees of freedom for the denominator, p-value (uncorr) – uncorrected p-value, ROI Left and ROI Right – region of interest in the left and right hemisphere
